# The Feasibility of a Machine Learning Approach in Predicting Successful Ventilator Mode Shifting for Adult Patients in the Medical Intensive Care Unit

**DOI:** 10.3390/medicina58030360

**Published:** 2022-03-01

**Authors:** Kuang-Hua Cheng, Mei-Chu Tan, Yu-Jen Chang, Cheng-Wei Lin, Yi-Han Lin, Tzu-Min Chang, Li-Kuo Kuo

**Affiliations:** 1 Graduate Institute of Clinical Medicine, College of Medicine, National Taiwan University, Taipei 10002, Taiwan; 2 Department of Critical Care Medicine, MacKay Memorial Hospital, Taipei 10449, Taiwan; 3 Department of Respiratory Therapy, MacKay Memorial Hospital, Taipei 10449, Taiwan; a0202@mmh.org.tw (M.-C.T.); yuchen5151@gmail.com (Y.-J.C.); 4 Software Product Center, Wistron Corporation, New Taipei City 22175, Taiwan; cwsralin@gmail.com (C.-W.L.); Irene_Lin@wistron.com (Y.-H.L.); Vicky_Chang@wistron.com (T.-M.C.); 5 Department of Medicine, Mackay Medical College, New Taipei City 25243, Taiwan

**Keywords:** critical care, decision support techniques, machine learning, respiratory insufficiency, ventilator weaning

## Abstract

*Background and Objectives*: Traditional assessment of the readiness for the weaning from the mechanical ventilator (MV) needs respiratory parameters in a spontaneous breath. Exempted from the MV disconnecting and manual measurements of weaning parameters, a prediction model based on parameters from MV and electronic medical records (EMRs) may help the assessment before spontaneous breath trials. The study aimed to develop prediction models using machine learning techniques with parameters from the ventilator and EMRs for predicting successful ventilator mode shifting in the medical intensive care unit. *Materials and Methods*: A retrospective analysis of 1483 adult patients with mechanical ventilators for acute respiratory failure in three medical intensive care units between April 2015 and October 2017 was conducted by machine learning techniques to establish the predicting models. The input candidate parameters included ventilator setting and measurements, patients’ demographics, arterial blood gas, laboratory results, and vital signs. Several classification algorithms were evaluated to fit the models, including Lasso Regression, Ridge Regression, Elastic Net, Random Forest, Extreme Gradient Boosting (XGBoost), Support Vector Machine, and Artificial Neural Network according to the area under the Receiver Operating Characteristic curves (AUROC). *Results*: Two models were built to predict the success shifting from full to partial support ventilation (WPMV model) or from partial support to the T-piece trial (sSBT model). In total, 3 MV and 13 nonpulmonary features were selected for the WPMV model with the XGBoost algorithm. The sSBT model was built with 8 MV and 4 nonpulmonary features with the Random Forest algorithm. The AUROC of the WPMV model and sSBT model were 0.76 and 0.79, respectively. *Conclusions*: The weaning predictions using machine learning and parameters from MV and EMRs have acceptable performance. Without manual measurements, a decision-making system would be feasible for the continuous prediction of mode shifting when the novel models process real-time data from MV and EMRs.

## 1. Introduction

The opportune weaning from the mechanical ventilator (MV) after acute respiratory failure prevents not only the jeopardy of premature weaning and extubation failure but also the risk of ventilator-associated pneumonia, vocal cord injury, tracheomalacia, and post-extubation laryngeal edema after prolonged intubation in the intensive care units (ICU) [1,2,3,4]. In many hospitals, the MV is initiated with volume-controlled ventilation (VCV) or pressure-controlled ventilation (PCV) modes (full support mode), and the readiness of successful weaning usually is determined with the physician-driven or protocol-based screening of weaning parameters. Many parameters derived from lung mechanics and respiratory patterns have been proposed for the prediction of successful weaning, including airway occlusion pressure 0.1 s (P 0.1), maximal inspiratory pressure (MIP), rapid-shallow breathing index (RSBI), and CROP (dynamic compliance, respiratory rate, oxygenation, maximum inspiratory pressure) index, etc. [5]. When the weaning parameters are acceptable, MV support may be reduced to partial support modes such as pressure support ventilation (PSV), synchronized intermittent mandatory ventilation, and continuous positive airway pressure ventilation (CPAP). After patients tolerate partial support modes, the international consensus recommended a spontaneous breathing trial (SBT) with T-piece (T-P) breathing or lower levels of pressure support 30 min to determine whether adult critical-ill patients can be successfully extubated [6].

The factors governing the weaning are multifactorial and interactional. Clinicians may be not able to notice the multidimensional factors effectively and initiate the weaning screening efficiently. Most of the weaning parameters require manual measurements with disconnecting the MV which increases the workload and risk of the air-born infection to therapists. And a single weaning parameter rarely provides sufficient accuracy to predict weaning outcomes [7]. Computer-aid decision-making systems may avoid human error and delay. Recent machine learning techniques are considered for timely and reliable weaning prediction. In predicting successful extubation in mechanically ventilated patients with the Artificial Neural Network (ANN), the area under the receiver operating characteristic curves (AUROC) was better than the traditional weaning parameter RSBI (0.83 vs. 0.66) [8]. In a recent review, there were only five research applying machine learning techniques in predicting successful weaning, and all training sets were small (8~179 patients). The model parameters were selected from demographics, vital signs, and ventilator data [9]. However, nonpulmonary factors such as serum hemoglobin and creatinine may also affect the outcome of MV weaning [10,11,12]. There was no study focused on predicting successful ventilator mode shifting from full support mode, partial support mode, and SBT.

The readiness of MV weaning was assessed daily by the ICU physicians and respiratory therapists in our hospital. The weaning parameters (tidal volume, maximal inspiratory/expiratory pressure, and RSBI) were screened before mode shifting. To improve our weaning process, we hypothesized that prediction models using machine learning techniques with parameters from the MV and nonpulmonary parameters could provide better predictions of successful ventilator mode shifting than traditional weaning parameters.

## 2. Materials and Methods

### 2.1. Study Subjects

This study was a retrospective analysis using data mining and supervised machine-learning methods based on a large electric database. The study protocol was approved by the Institutional Review Board of the MacKay Memorial Hospital (18MMHIS063e, approval on 30 July 2018).

#### 2.1.1. Data Source

Mackay Memorial hospital is a tertiary medical center with 120 ICU beds. The data of clinical diagnoses, age, gender, vital signs, laboratory data, arterial blood gas, vital signs, patients’ diagnoses, demographics, and prescribed medications were stored in the electronic medical records (EMRs) system. The MV setting and monitoring parameters were recorded and uploaded automatically by the Vital Info Portal Gateway (Maya International Company, Ltd., Taipei, Taiwan) per hour. The data of EMR and MV from three medical ICU (39 beds) between April 2015 and October 2017 were analyzed retrospectively.

#### 2.1.2. Participants

Patients older than 19 years old who required invasive mechanical ventilator support in the ICU were included. Exclusion criteria included patients who expired during the ICU course or were readmitted to the ICUs within 14 days because critically ill patients may have few attempts of MV weaning. Eventually, 1483 patients were enrolled, and the data were analyzed from the first day of ICU admission.

In our cohort, the readiness of MV weaning was assessed daily by the ICU physicians and respiratory therapists. The weaning parameters (tidal volume, maximal inspiratory/expiratory pressure, and RSBI) were screened before SBT. The MV setting and measurement data were captured, stored, and uploaded automatically by Vital Info Portal Gateway per hour. The MV alarm and setting changes were recorded and uploaded immediately into the database.

### 2.2. Predicting Models

#### Outcomes

In the retrospective dataset, weaning of MV mostly was conducted through a sequence of full-support MV mode shifting. The primary outcomes were successful events of the MV mode shifting. Full support modes comprised of VCV and PCV. Partial support modes included PSV and CPAP. A successful shifting from full support to partial support mode was defined arbitrarily as a PSV or CPAP followed by a T-P trial regardless of the duration and pressure levels of PSV. The unsuccessful shift was defined when the PSV/CPAP trial was not followed by a T-P trial (meaning shifting back to full-support mode).

A T-P trial of 30–120 min can predict 75.9% of patients who remain extubated for 48 h [13]. Therefore, a successful shifting from partial support mode to T-P was defined as T-P duration longer than 2 h in the study. An unsuccessful shift meant the T-P switching back to a full-support mode in less than 2 h.

### 2.3. Candidate Predictors

The candidate predictors for MV weaning were classified into five categories: demographic data, arterial blood gas, laboratory data, vital sign, and ventilator information. Table 1 lists the potential predictors during ICU admission and the range for discarded outliers.

Several predictors within a specific time window were further derived statistically, including average, variance, median, slope, coefficient of variation (CV). The Slope was calculated from the fitted simple line regression. All derivatives were named with the following rule: PredictorName_Statistics_TimeWindow.

### 2.4. Missing Data

For values missing at random in the dataset, the index (NA indicator) was designed to calculate whether the data were recorded or not within the time window.

### 2.5. Data Mining

The training sets randomly selected from 70% of the cleaned dataset were used to train the prediction model, and the residual 30% of the cleaned dataset was used to evaluate the performance of the prediction model.

### 2.6. Modeling

The first model (weaning probability of mandatory ventilation, WPMV) was built to predict successful shifting from full-support to partial-support modes. The second model (successful SBT, sSBT) was built to predict the shifting from partial-support mode to successful T-P trials.

We tried several classification algorithms to fit the models, including Lasso Regression, Ridge Regression, Elastic Net, Random Forest, Extreme Gradient Boosting (XGBoost), Support Vector Machine (SVM), and Artificial Neural Network (ANN).

Feature selection is an essential procedure in building a machine learning classifier. We selected the features in three steps. First, a *t*-test was used to choose significant predictors from candidate predictors. Second, variable importance was measured in XGBoost and Random Forest by information gain and mean decrease in Gini separately. Then the top 30 predictors were extracted. Third, combining the two subsets of candidate predictors, we utilized backward elimination to delete predictors that perform worse.

The performance of binary classification models was determined by drawing the receiver operating characteristic (ROC) curves and analyzing the indicators of the confusion matrix. The algorithm with had the largest area under the ROC (AUROC) would be chosen for the final model.

**Figure 1:**
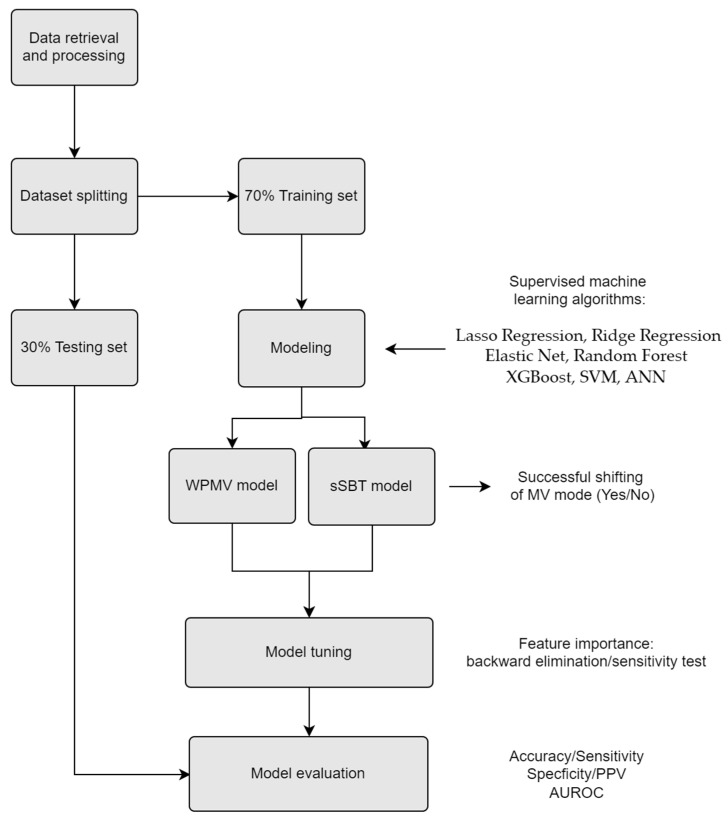
The workflow of our study. XGBoost: Extreme Gradient Boosting, SVM: Support Vector Machine., ANN: Artificial Neural Network. WPMV: weaning probability of mandatory ventilation. sSBT: successful spontaneous breathing trial. MV: mechanical ventilator. PPV: Positive Predictive Value. AUROC: area under the receiver operating characteristic curves.

All analyses were carried out in R software version 3.4.1 (R Core Team, Vienna, Austria) with XGboost and random Forest packages. The workflow of the analysis is illustrated in Figure 1.

## 3. Results

### 3.1. Participants and Outcomes

A total of 1483 patients were enrolled, and the data were analyzed from the first day of ICU admission. The dataset came from elderly patients (mean age 66.9 years old) with multiple comorbidities, and infection was the most frequent etiology of their acute respiratory failure (39.8%). The successful rate of weaning from MV for more than 5 days was 77% in the cohort. The demographic characteristics of the enrolled patients are outlined in Table 2.

For the WPMV prediction model, a total of 2153 events of full-support shifting to partial-support mode were randomly assigned to the training set (*n* = 1510) and test set (*n* = 643). The 1275 events (59.2%) were found successful.

For the sSBT model, there were 3132 events of partial mode shifting to T-P randomly assigned to the training set (*n* = 2201) and test set (*n* = 931). A total of 1520 successful events (48.5%) with T-P longer than 2 h were found.

### 3.2. Model Performance

**Figure 2:**
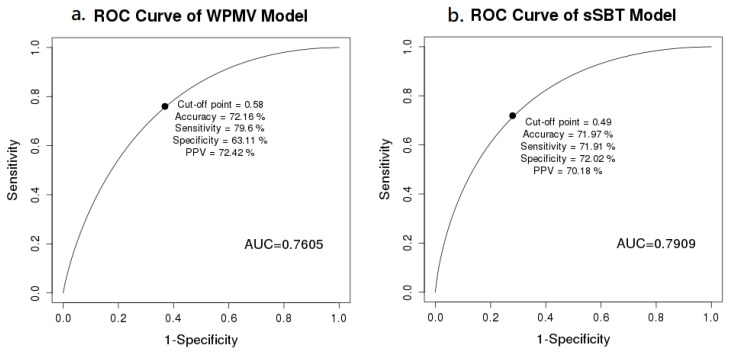
Receiver operating characteristic curve of the weaning probability of mandatory ventilation prediction model (**a**) and successful spontaneous breathing trial prediction model (**b**).

The performances of the binary classification models were determined by the ROC and confusion matrix (Figure 2). The XGBoost algorithm had the largest AUROC 0.76 applied for WPMV, and Random Forest with the largest AUROC 0.79 was chosen for sSBT model. The AUROC of the WPMV model and the sSBT model were 0.76 and 0.79, respectively. At the cut-off value of 0.58 for best accuracy, the WPMV model had the sensitivity 79.6%, specificity 63.1%, and accuracy 72.2%. The sSBT model had the best accuracy (80%) at cut-off value of 0.49 (sensitivity 71.9%, specificity 72%).

### 3.3. Predictor Importance

**Figure 3:**
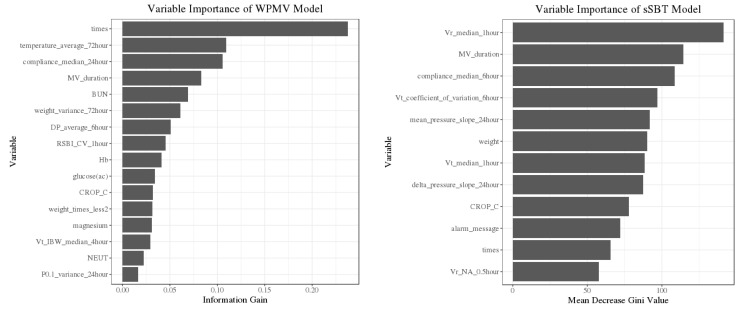
Importance ranking of predictors in WPMV and sSBT model. (WPMV: weaning probability of mandatory ventilation. sSBT: successful spontaneous breathing trial. times: number of ventilator mode shifting. MV: mechanical ventilator; BUN: blood urea nitrogen. DP: diastolic pressure. RSBI: rapid-shallow breathing index. CROP: Compliance, Respiratory Rate, Oxygenation, and Pressure index. Vt: tidal volume. IBW: ideal body weight. Vr: ventilation rate).

The importance of predictors was shown from high to low importance according to the information gain (WPMV model) and mean decrease Gini (sSBT model) in Figure 3. The characters of selected features in successful and unsuccessful events were outlined in Table 3. Several predictors have no significant difference between successful and unsuccessful events. However, the insignificant predictors still provide important scores in XGBoost and Random Forest. When insignificant predictors were removed from the models, the AUROC of the WPMV and sSBT models were reduced to 0.73 and 0.77.

Among the 16 selected predictors in the WPMV model (3 MV and 13 nonpulmonary parameters), many of them related to clinical conditions e.g., body temperature, body weight, blood urea nitrogen, hemoglobin, and the ratio of neutrophils. The average body temperature in the previous 72 h was the most important nonpulmonary feature. In the sSBT model, 12 predictors were chosen (8 MV and four nonpulmonary parameters), and the most important one was “vr_median_1hour” which indicated the median of ventilation rates within 1 h. The many of selected features highlighted that the trend of respiratory mechanics over a specific time window had greater predictive value than at a single time point, e.g., “median of compliances with 6 h”, “median of tidal volumes within 1 h”, and” slope of mean airway pressure within 24 h”.

For sensitivity analysis, the predictors in Table 3 were divided into two groups: ventilator (MV) and non-ventilator (nonpulmonary). When non-MV predictors were removed, the AUROC of the WPMV and sSBT models was reduced to 0.66 and 0.76. On the other hand, the AUROC of the WPMV and sSBT models were 0.74 and 0.65 if the MV predictors were removed from the models. The results indicated that non-MV predictors were more dominant than MV predictors in the WPMV, while MV predictors were more important than non-MV predictors in the sSBT model.

The 1275 events (59.2%) were found successful in the 2153 events of full-support shifting to partial-support mode in the WPMV model. For the sSBT model, 1520 successful events (48.5%) were found in the 3132 partial-support shifting to T-P. The XGBoost and Random Forest were found to have the largest AUROC and chosen for the WPMV and sSBT model accordingly.

XGBoost and Random Forest can measure the importance of features by information gain and mean decrease in Gini separately. Table 3 lists the selected predictors in ranking from high to low importance according to the information gain (WPMV model) and mean decrease Gini (sSBT model). Among the 16 selected predictors in the WPMV model (three ventilatory and 13 clinical parameters), the number of transitions events from full to partial-support mode during weaning was the most important predictor. In the sSBT model, 12 predictors were chosen (eight ventilatory and four clinical parameters), and the most important one was “vr_median_1hour” which indicated the median of ventilation rate within 1 h.

## 4. Discussion

With the integration of machine learning algorithms and variables from the MV and EMRs, our prediction models (WPMV and sSBT) showed acceptable discrimination for predicting successful MV mode shifting. By sSBT model, the performance in predicting of discontinuing MV support is better than traditional parameter RSBI, where AUROC was 0.69 in a previous review article [14].

The strength of our prediction models includes the large number of participants and the hourly data input from MV. Therefore, we could find that the novel predictor “median of ventilation rate within 1 h” is the most important feature in sSBT model. In previous studies, the AUROC of an Artificial Neural Network (ANN) model was 0.83 for predicting successful weaning and extubation [8] and 0.942 for difficult weaning [15]. For predicting successful SBT, the accuracy was 81–86.7% by Support Vector Machine (SVM) [16,17]. However, all the models need parameters of the respiratory pattern in 30-min SBT. The models cannot help the early initiation of MV mode shifting before SBT. Our dataset was larger than the previous study used with ANN and SVM. Our models were trained with time-series variables from MV and EMR since the first day of ICU admission. Thus, it may be feasible to continuously predict successful MV mode shifting when a platform would be built with our models to process real-time variables from MV and EMRs simultaneously.

The most common processes of weaning were conducted by T-P following PSV, and the practice may be different from other hospitals. However, our studied population and weaning outcomes were comparable to a recent study [18]. The success rate of the first SBT in our cohort was 75%, which was similar to the previous report (79% in J-M. Boles et al.) [6]. Thus, the performance of the weaning protocol in our hospital was comparable to other institutions.

Compared with traditional algorithms such as linear regression, XGBoost, and Random Forest, they are more reliable in discovering non-linear relationships from our data [19,20], and can measure the importance of features. Among the nonpulmonary parameters in the WPMV model, blood urea nitrogen, hemoglobin, and fluid balance have contributions consistent with previous reports [21,22,23]. The glucose, magnesium level, and average diastolic blood pressure are first reported, and their clinical significances in the weaning process need further investigation.

**Figure 4:**
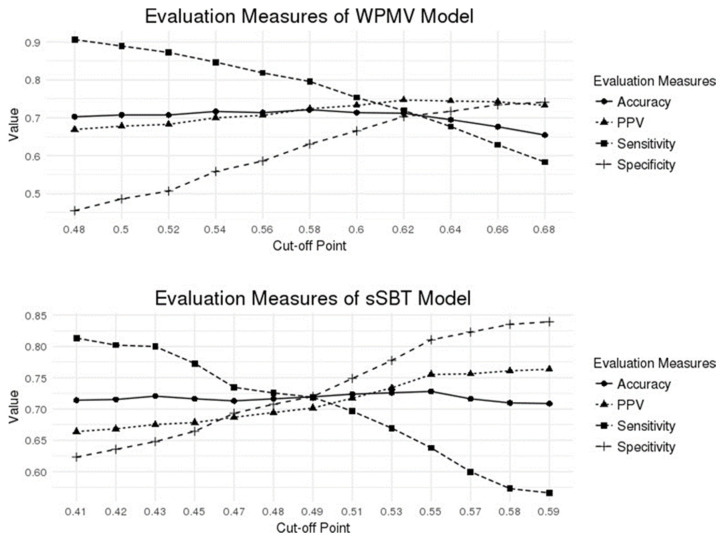
Evaluation measures under different cut-off points of weaning probability of mandatory ventilation model and successful spontaneous breathing trail model.

The current cut-off value was targeted at the best accuracy. In real-world practice, the value selection can depend on the intention of the physicians. For early weaning, the cut-off value should be chosen for the highest sensitivity. Then, our WPMV model would identify the 90% of patients ready for partial support at a cut-off value of 0.48, while the sSBT model detects the 80% of patients who can tolerate T-P 2 h at a cut-off value of 0.41, as shown in Figure 4. However, the positive predictive value (PPV) at these cut-off values was around 70%, and further confirmation tests may be performed to avoid premature weaning in high-risk populations

## 5. Limitation

First, this single-center designed project may have the risk of overfitting and limit the generalization ability of the predictive model to other ICUs as the weaning practice may differ in other institutions [24]. Second, some parameters were dependent on the clinicians’ practice rather than patient characteristics (e.g., frequency of ABG, blood pressure, laboratory tests, weights recording, MV alarm and tidal volume setting, etc.). As a result, some parameters, such as Vt_weight_median_4hour, weight_variance_72hour, count of the alarm message, and DP_average_6hour may be affected in the retrospective study.

Third, the amount of clinical data collected for 2 years in a single hospital may not be sufficient to train the models because of complicated situations in medical ICUs. Our model excluded expired and re-admitted patients’ data due to the low possibility of successful weaning. However, this may hinder the models to predict weaning in the real-world setting.

Fourth, extubation is an important issue after successful weaning from the ventilator. In our retrospective dataset, the time of extubation was not recorded and the current study cannot address the prediction of successful extubation. The cause of resuming MV support after a T-P trial could not distinguish SBT failure from extubation failure. A further prospective study with a better information recording is mandatory for the issue of successful extubation.

Finally, the accuracy of our current WPMV and sSBT models are 72.2% and 71.97%. The values may fulfill the minimal requirement for clinical practices. In theory, transfer learning techniques and multi-center data would improve the predictive ability of our models applying in other institutions.

## 6. Conclusions

**Figure 5:**
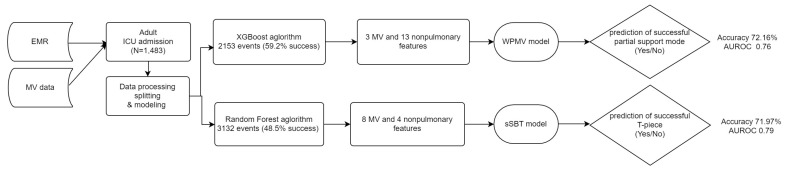
The illustration of our tangible results.

This is the first study focused on predicting successful MV mode shifting from full support, partial support to SBT with machine learning techniques. Our study highlighted the accurate prediction of MV weaning using multiple domains of clinical parameters and hourly input of MV variables with novel statistics (average/variance/median/slope) of pulmonary mechanics. The prediction models using data from EMR and MV require no manual measurement of weaning parameters and have better AUROC than traditional RSBI. Nonpulmonary features related to body temperature, weight, blood urea nitrogen, hemoglobin, and the ratio of neutrophils are important for successful partial support ventilation. The trend of pulmonary mechanics hours before SBT is crucial in predicting a successful T-P trial. Further larger multi-center training datasets with transfer machine learning techniques may improve the performance of these classification models. A decision-making system will be feasible for opportune weaning when the novel models process real-time data from MV and EMRs continuously. The tangible results of our study are demonstrated in Figure 5.

## Figures and Tables

**Table 1:** List of candidate predictors extracted from the electronic medical records.

Category	Candidate Predictors (Abbreviation Used throughout This Study)	Range of the Outliers (Unit)
**Ventilator information**	Tidal Volume (Vt)	Vt < 1 or Vt > 1260 (mL)
	Ventilation Rate (Vr)	Vr < 1 or Vr > 50 (breath per minute)
	airway occlusion pressure (P0.1)	nil
	Dynamic Compliance	nil
	Mean Pressure	mean_pressure < 0.1 or >28.2 (cm H_2_O)
	Resistance	Resistance < 0 or >30 (cm H_2_O/L/s)
	fraction of inspired oxygen (FiO_2_)	FiO_2_ < 21% or FiO_2_ > 100%
	positive end expiratory pressure (PEEP)	nil
	alveolar oxygen partial pressure (PAO_2_)	nil
	number of transitions from full to partial-support mode during weaning (numbers of WPMV)	nil
	numbers of transitions from partial-support mode to full-support mode or T-P during weaning (numbers of SBT)	nil
	Accumulated total time of using mechanical ventilator (MV_duration)	nil
	Numbers of alarm message per minute on MV (alarm_message)	nil
**Arterial** **Blood Gas**	Arterial carbon dioxide partial pressure (PaCO_2_)	nil
	arterial oxygen partial pressure (PaO_2_)	PaO_2_ < 23.1 or >480 (mmHg)
	arterial oxygen saturation (SaO_2_)	SaO_2_ < 39.7 or >100 (%)
	base excess (BE)	BE < −27.7 or BE ≥ 93.5 (mEq/L)
	potential of hydrogen (pH)	nil
**Laboratory** **data**	Albumin, Blood Urea Nitrogen (BUN)	nil
	Creatinine	nil
	Glucose	nil
	Hemoglobin (Hb)	nil
	white blood cell (WBC)	nil
	differential count: Neutrophil (NEUT)	nil
**Vital Sign**	Glasgow Coma Scale (GCS)	GCS < 3 or GCS > 15
	Diastolic Blood Pressure (DP)	DP ≤ 0 or DP ≥ 150 (mmHg)
	Systolic Blood Pressure (SP)	SP ≤ 0 or SP ≥ 268 (mmHg)
	Pulse	Pulse < 30 or > 235 (beat per minute)
	Temperature	Temperature < 32 or >42 (°C)
**Demographic data**	Age	nil
	Gender	nil
	Patient’s weight (weight)	nil

**Table 2:** Characteristics of the study population.

**Characteristic**	**Statistical Analysis**
General	
Gender	
male	927 (62.5%)
female	556 (37.5%)
Age	66.88 ± 15.42
APACHE II score	21 (17–27)
In-ICU days	7 (3–12)
MV day	3.91 (1.67–8.73)
Count of shifting between full mode and partial mode	1 (1–2)
Count of shifting between partial mode and SBT	1 (1–2)
**Diagnostics/Diseases**	
Cause of respiratory failure	
Pulmonary edema	18 (1.2%)
Systolic congestive heart failure	460 (29.6%)
Acute myocardial infraction	63 (4.0%)
Chronic obstructive pulmonary disease	43 (2.8%)
Asthma	33 (2.1%)
Pneumonia	289 (18.6%)
Bronchopneumonia	20 (1.3%)
Urinary tract infection	77 (4.9%)
Sepsis	505 (32.5%)
Toxicity of carbon monoxide	40 (2.6%)
**Comorbidity**	
Diabetes mellitus	368 (23.7%)
Brain related (infarction/hemorrhage)	501 (32.2%)
Kidney related (ESRD, acute/chronic kidney disease)	149 (9.6%)
Liver related (alcoholic cirrhosis, HBV/HCV cirrhosis)	95 (6.1%)
Lung related (tuberculosis)	2 (0.1%)
Malignancy (all types)	133 (8.5%)

General characteristics were analyzed by patient number; diagnostic characteristics were analyzed by admission number. Continuous data with normal distribution are expressed as mean and standard deviation, non-normal continuous data are expressed as median [interquartile range], and discrete data are expressed as count and percentage. APACHE: Acute Physiology and Chronic Health Evaluation. ICU: intensive care unit. MV: mechanical ventilator. SBT: spontaneous breathing trial. ESRD: end stage renal disease. HBV: hepatitis B virus. HCV: hepatitis C virus.

**Table 3:** Summary of selected predictors of the developed model with successful and unsuccessful events.

Predictors (Unit)	Successful Event	Unsuccessful Event	*p*-Value
WPMV model			
Numbers of WPMV (count)	1 (1–2)	2 (1–4)	<0.01
temperature_average_72hour (°C)	36.99 ± 0.61	37.15 ± 0.65	<0.01
compliance_median_24hour (mL/cm H_2_O) ^M^	52.65 ± 28.20	43.37 ± 24.41	<0.01
MV_duration (hour)	48.0 (21.1–98.6)	88.49 (40.4–164.2)	<0.01
Blood Urea Nitrogen (mg/dL)	38.70 ± 32.24	47.87 ± 34.03	<0.01
weight_variance_72hour (kg)	0.15 ± 2.67	0.03 ± 0.15	0.357
DiastoicPressure_average_6hour (mmHg)	67.50 ± 11.95	65.67 ± 12.07	<0.01
RSBI_CV_1hour (breaths/min/mL) ^M^	0.66 ± 0.43	0.72 ± 0.47	0.017
Hemoglobin (g/dL)	10.47 ± 2.00	10.33 ± 1.94	0.137
Glucose (mg/dL)	166.27 ± 87.57	166.31 ± 103.31	0.995
CROP_C	0.02 ± 0.02	0.01 ± 0.02	0.01
numbers of weight records < 2 before partial mode (%)	212 (16.6)	103 (11.7)	0.002
magnesium (mg/dL)	2.14 ± 0.48	2.19 ± 0.47	0.076
Vt_weight_median_4hour (mL/kg) ^M^	8.39 ± 1.88	8.50 ± 2.18	0.256
Neutrophil (%)	80.62 ± 10.66	80.33 ± 10.53	0.5598
P0.1_variance_24hour (cm H_2_O) ^M^	1.08 ± 3.78	2.25 ± 12.16	0.136
sSBT model			
Vr_median_1hour (breaths/min) ^M^	19.85 ± 8.00	21.07 ± 10.49	0.002
MV_duration (hour)	49.08 (20.0–109.2)	79.14 (35.2–154.2)	<0.01
compliance_median_6hour (mL/cm H_2_O) ^M^	67.58 ± 39.60	59.04 ± 50.17	<0.01
Vt_CV_6hour (mL) ^M^	0.40 ± 0.25	0.39 ± 0.22	0.253
mean_pressure_slope_24hour (cm H_2_O) ^M^	−0.00 ± 0.01	0.00 ± 0.04	0.183
weight (kg)	60.87 ± 14.19	61.08 ± 14.44	0.69
Vt_median_1hour (mL) ^M^	415.93 ± 186.20	417.13 ± 189.43	0.868
delta_pressure_slope_24hour (cm H_2_O) ^M^	−0.00 ± 0.02	−0.01 ± 0.18	0.1
CROP_C	0.03 ± 0.08	0.02 ± 0.04	0.213
alarm_message (count) ^M^	0 (0–5)	4 (0–13)	<0.01
Numbers of SBT (count)	1 (1–2)	2 (1–3)	<0.01
No Vr change in 0.5hour (%)(Vr_NA_0.5hour = 1) ^M^	973 (64.0)	496 (30.8)	<0.01

Numbers of WOMV, MV_duration, numbers of SBT and alarm_message are expressed as median (interquartile range), the rest are expressed as mean and standard deviation. Continuous predictors and discrete predictors are expressed as counts and percentages. ^M^ indicates the predictors in MV group for sensitivity analysis. Vt: tidal volume, Vr: respiratory rate, RSBI: vr/vt. Vt_weight: vt/ideal body weight, delta_pressure: peak pressure—PEEP. CROP_C: dynamic compliance/Vt * PaO_2_/PAO_2_.

## Data Availability

All the data supporting our findings are available from the corresponding author upon reasonable request.
